# Is Endoscopy Really Necessary for Placing Intragastric Balloons?

**DOI:** 10.1007/s11695-017-2812-5

**Published:** 2017-07-10

**Authors:** Elisabeth M.H. Mathus-Vliegen

**Affiliations:** Academic Medical Centre, University of Amsterdam, Meibergdreef 9, 1105 AZ Amsterdam, the Netherlands

**Keywords:** Intragastric balloons, Intragastric balloon therapy, Obesity, Weight loss treatment, Endoscopy, Adverse events

## Abstract

**Background:**

Gastric balloons for weight loss have historically been placed after a screening endoscopy. However, the utility and yield of these endoscopies has not been studied. Therefore, we wanted to evaluate the utility of screening endoscopy and to assess patients who had balloons placed without endoscopy.

**Methods:**

Data was collected on two cohorts. Cohort 1 consisted of patients who had a screening endoscopy prior to or upon balloon placement. Cohort 2 consisted of patients who were followed after having a balloon placed under fluoroscopic guidance without endoscopy. Balloon intolerance and findings on removal endoscopy were assessed in both cohorts.

**Results:**

In cohort 1 (*n* = 253), two patients had severe symptoms on history; balloon placement was contraindicated based on screening endoscopy findings. Eleven patients with a history of hiatal hernia and the presence of severe belching demonstrated an insignificant hiatal hernia on endoscopy. In cohort 2 (*n* = 50), all patients had an unremarkable history. Three previously asymptomatic patients had balloon intolerance and one was found to have a 4-cm hiatal hernia and oesophagitis upon balloon removal. Out of 194 patients, 25 were either intolerant to the balloon or had relevant findings on removal endoscopy. Findings on screening endoscopy did not correlate with balloon intolerance or findings on removal endoscopy.

**Conclusion:**

These results demonstrate that a careful history can identify patients who may have contraindications for balloon therapy and that balloons can be placed safely after taking a careful history without screening endoscopy. Screening endoscopy may not be useful in predicting balloon intolerance or potential complications.

## Introduction

The global increase in obesity warrants adequate prevention programmes and effective treatment strategies [1]. Evidence-based guidelines adopt a stepwise approach, consisting of lifestyle changes such as energy restriction, physical exercise and behaviour changes, followed by pharmacotherapy [2–4].

Bariatric surgery is only considered for individuals with a BMI ≥40 kg/m^2^ or a BMI ≥35 kg/m^2^ with at least one obesity-related comorbidity [3, 4]. However, only 1–2% of individuals who qualify for surgery elect to have it, mostly due to fear of complications [5]. Endoscopic therapy—including intragastric balloons—has emerged as an option for the moderately obese, closing the therapeutic gap between modestly effective drug therapy and bariatric surgery [6, 7].

Historically, intragastric balloons have required endoscopy and sedation. The oesophagus and stomach were typically assessed for contraindications either with a screening endoscopy or concomitant to device placement. However, endoscopy is not without risks in obese patients.

We first correlated endoscopic findings upon balloon placement to the patient history and to endoscopic findings upon balloon removal and then evaluated what aspects of the patient history were most critical to assess. Using these results, we studied the outcome of patients who had an unremarkable history in whom the balloon was placed only with fluoroscopy.

In order to accomplish these aims, we first evaluated patients who had their balloons inserted after a screening endoscopy or under direct endoscopic visualisation (cohort 1). Subsequently, in cohort 2, we followed patients who had balloons inserted under fluoroscopy without endoscopy after a detailed medical history.

## Materials and Methods

### Subjects

All patients referred for intragastric balloon therapy were screened using a detailed medical history. A detailed history was taken focused on upper gastrointestinal symptoms including heartburn, belching, nausea, vomiting, epigastric pain/dyspepsia and the relationship of these symptoms to diet and posture, a history of peptic ulcer disease, the presence of a hiatal hernia, medication use, surgery and type of surgery and a family history of gastrointestinal neoplasia. Those who reported to have a hiatal hernia first underwent an X-ray examination with contrast. To guarantee a consistent reporting of the history, only patients who participated in previous clinical trials conducted by the author were included.

Endoscopy for positioning and removal of the balloon was performed under conscious sedation with midazolam while monitoring vital signs. In both cohorts, the balloons were removed with endoscopy.

#### Cohort 1

Three different balloons were placed in this series (Table 1). Both the Wilson-Cook balloons (Wilson-Cook Medical, Winston Salem, NC, USA) and the Ballobes balloon (DOT ApS, Rodovre, Denmark) were inserted after a screening endoscopy [8–10]. Balloons were placed immediately after removal of the endoscope. For the Orbera balloon (Apollo Endosurgery, Austin, TX, USA), first, the endoscopy was done and then the balloon was inserted and filled under direct endoscopic visualisation [11–15].

**Table 1:** Characteristics of balloons and patients studied in each cohort

Balloon	Filling	Period	Duration	Study design	Scientific interest and publication
Cohort 1					
Wilson-Cook	300 mL air	February 1984–April 1987	27 weeks	Open	Gastric biopsies for pathology and *Helicobacter pylori* [8]
Ballobes	500 mL air	February 1988–March 1989	36 weeks	Double-blind sham-controlled cross-over (7 sham-sham)	24-h pH measurements, manometry [9, 10]
Orbera	500 mL saline	April 1994–August 1996	52 weeks	Double-blind sham-controlled	24-h pH measurements, manometry [11–15]
Cohort 2					
Orbera	500 mL saline	July 1996–August 2002	27 weeks	Open	24-h pH measurements, manometry, gastric emptying [14–16]

#### Cohort 2

In this series, no endoscopy was performed but the diaphragm was visualised under fluoroscopy and the Orbera balloon positioned 10 cm below the diaphragm [14–16].

### Statistics

Descriptives are given as means and standard deviation (SD). Group characteristics were compared using Student’s *t* test or Mann-Whitney *U* test in case of a non-Gaussian distribution. Subgroups were compared using ANOVA with Bonferroni’s correction for multiple testing or Kruskal-Wallis in not normally distributed values. Chi square statistics with Fisher’s exact test when indicated were used to compare categorical values and risk differences and the odds ratio (OR) with 95% confidence intervals (CI) were calculated. The Newcombe-Wilson method was used to calculate the 95% confidence interval for the prevalence differences in baseline characteristics. A *p* value <0.05 was considered significant.

## Results

**Fig. 1:**
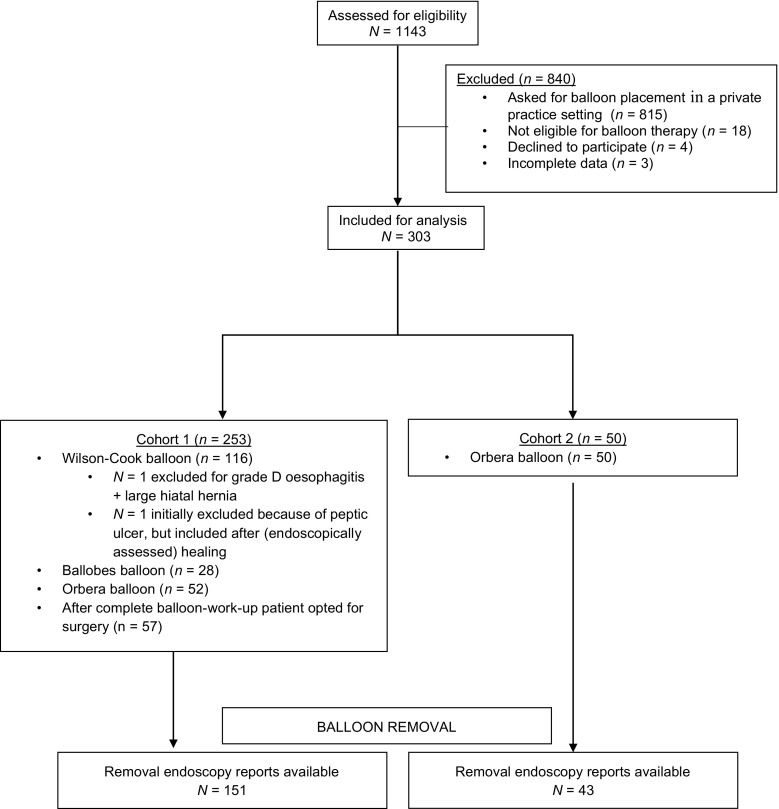
Flow diagram

A total of 1143 patients (216 males) were referred for intragastric balloon therapy of whom 303 (78 males) with a mean age 36.7 ± 9.8 years were considered eligible with a mean weight of 138.4 ± 27.4 kg and a mean BMI of 46.4 ± 7.8 kg/m^2^. A total of 840 patients were excluded, of which 815 requested balloon placement in a private practice setting (Fig. 1).

Of these 303 patients, 253 were part of cohort 1 and 50 were part of cohort 2. There were no differences between cohort 1 and cohort 2 in age, body weight and BMI (Table 2).

**Table 2:** Baseline characteristics by cohort

	Cohort 1	Cohort 2	*p* value
*n*	253 (70 M; 183 F)	50 (8 M; 42 F)	>0.05
Age (years)	36.4 ± 10.2 (range 15–70)	35.8 ± 6.8 (range 20–54)	>0.05
Weight (kg)	139.5 ± 26.7 (range 93.0–222.0)	140.0 ± 24.6 (range 101.1–206.0)	>0.05
BMI (kg/m^2^)	47.0 ± 7.7 (range 31.1–86.2)	46.0 ± 5.8 (range 33.8–61.7)	>0.05

### Cohort 1: Balloon Placement After Screening Endoscopy

Out of 253 patients, a reliable medical history was present in 250 and full endoscopic reports were available in 243 patients (Fig. 1). After the screening endoscopy, 57 patients elected for bariatric surgery and thus did not participate in balloon therapy. Of note, these patients underwent upper abdominal X-rays with contrast and thus allowed for comparison to endoscopic findings, specifically with regard to presence of hiatal hernia. One hundred fifteen patients had a Wilson-Cook balloon and 28 had a Ballobes balloon with confirmation of positioning by subsequent abdominal X-ray. In 52 patients, the Orbera balloon was inserted under direct endoscopic visualisation.

#### Findings on Screening History, Endoscopy and Radiography

On history, 85 (34%) of patients had diet-related heartburn with no position dependence and only 16 (6.4%) needed antacids, H_2_ blockers or PPIs; 27 (10.8%) had epigastric pain or dyspepsia and 13 (5.2%) had a known hiatal hernia (Table 3). On screening endoscopy, the only major findings were grade D oesophagitis combined with a large hiatal hernia in one patient and a peptic ulcer in another. Both patients complained of severe symptoms on history.

**Table 3:** Findings on history, screening endoscopy/balloon placement in cohort 1

Medical history (*N* = 250)	Present *n* (%)
• Heartburn	85 (34.0)
• Belching	16 (6.4)
• Nausea	12 (4.8)
• Vomiting	7 (2.8)
• Gastric pain/dyspepsia	27 (10.8)
• Known hiatal hernia	13 (5.2)
• History of peptic ulcer	9 (3.6)
Medication related to the stomach	
• No medication	234 (93.6)
• Antacids	8 (3.2)
• H_2_ receptor blockers	6 (2.4)
• Proton pump inhibitors	2 (0.8)
Surgical history (*N* = 250)	
• No surgery	187 (74.8)
• Gastric surgery	2 (0.8)
• Cholecystectomy	23 (9.2)
• Appendectomy	31 (12.4)
• Combined upper and lower gastrointestinal surgery	7 (2.8)
Screening endoscopy/balloon placement (*N* = 243)	
• Oesophagitis	12 (4.9)
○ Grade A	9
○ Grade B	2
○ Grade D	1
• Gastric erosions	17 (7.0)
• Gastritis	3 (1.2)
• Ulcer	1 (0.4)
• Hiatal hernia	11 (4.5)
• Biopsies *H*. *pylori* (*n* = 109)	21 (19.3)
• Biopsies acute/chronic inflammation (*n* = 109)	35 (32.1)

Only some aspects of the history correlated to endoscopic or biopsy findings (Table 4). Severe nausea and vomiting were associated with the endoscopic finding of a peptic ulcer. Belching and the history of having a hiatal hernia correlated with the endoscopic presence of a hiatal hernia, and heartburn was associated with chronic gastritis upon biopsy.

**Table 4:** Correlations between symptoms and endoscopic findings and findings upon biopsy

Symptom	Ulcer on endoscopy % (*n*/*N*)	No ulcer on endoscopy % (*n*/*N*)	Chi square with Fisher’s exact test	Difference (95% CI)	OR (95% CI)
Severe nausea	100 (1/1)	4.6 (11/241)	0.05	95.4 (16.0/97.4)	n.a.
Severe vomiting	100 (1/1)	2.5 (6/241)	0.029	97.5 (18.1/98.9)	n.a.
	HH on endoscopy % (*n*/*N*)	No HH on endoscopy % (*n*/*N*)			
Belching	33.3 (4/12)	5.2 (12/230)	0.005	28.1 (8.3/55.8)	9.08 (2.39/34.47)
History of HH	16.7 (2/12)	3.5 (8/230)	0.05	13.2 (0.8/41.4)	5.55 (1.04/29.60)
	Chronic gastritis on pathology % (*n*/*N*)	No chronic gastritis on pathology % (*n*/*N*)			
Heartburn	42. 9 (15/35)	23.3 (17/73)	0.045	19.6 (1.1/37.8)	2.47 (1.04/5.85)

*HH* hiatal hernia, *pathology* pathology on biopsy specimen, *n.a.* not applicable

In 57 patients who opted for surgery and not for balloon treatment, both an endoscopy and X-ray with contrast were performed. Six (11%) patients reported to have a hiatal hernia. In one patient, this was confirmed by both endoscopy and X-ray. In the other five, a small, insignificant hiatal hernia was seen only on X-ray and not on endoscopy.

#### Findings on Balloon Removal

Seven patients did not receive a balloon at all and were excluded from this dataset (Table 1). Another 37 had their balloons removed elsewhere without providing a full endoscopic report, thus leaving 151 patients with follow-up endoscopy after balloon removal.

Four (2.6%) patients did not tolerate the balloon and upon endoscopy, no abnormalities were found, and two (1.3%) patients reacted favourably to adjustment in balloon volume (Table 5). Another four (2.6%) did not tolerate the balloon and were found to have either grade D (*n* = 2), grade C (*n* = 1) or grade B (*n* = 1) oesophagitis which rapidly healed after removal of the balloon. Eleven (7.3%) patients had ulcers: seven were asymptomatic, two had symptoms of balloon intolerance not necessitating balloon removal and two presented with bleeding ulcers. Neither the patient history nor the findings on screening or placement endoscopy correlated to these adverse events.

**Table 5:** Findings upon balloon removal in cohorts 1 and 2

Findings	Cohort 1 *N* = 151 present *n* (%) [balloon]	Cohort 2 *N* = 43 present *n* (%)	Difference in proportions with 95% CI
Oesophagitis	12 (7.9)	4 (9.3)	−1.4 (−19.2/8.7)
• Grade A	8 (5.3)	0	5.3 (−8.5/12.2)
• Grade B	1 (0.6) [O]	3 (7.0)	−6.3 (−23.1/0.7)
• Grade C	1 (0.6) [O]	1 (2.3)	−1.7 (−16.5/3.5)
• Grade D	2 (1.3) [both O]	0	1.3 (−12.1/6.5)
Gastric erosions	18 (11.9)	0	11.9 (−2.5/20.4)
Gastritis	1 (0.7)	0	0.7 (−12.8/5.4)
Ulcer	11 (7.3) [WC = 9, B = 2]	0	7.3 (−6.7/14.7)
Hiatal hernia	6 (4.0)	3 (7.0)	−3.0 (−19.9/5.2)
Intolerance without specific findings	4 [WC = 1, B = 1, O = 2]	2 (4.7)	−2.0 (−18.0/4.9)

For the relevant findings in cohort 1, the balloon type is listed in brackets. In cohort 2, all balloons were Orbera balloons

*WC* Wilson-Cook balloon, *B* Ballobes balloon, *O* Orbera balloon, *CI* confidence intervals

On three occasions, balloon removal itself resulted in lacerations at the gastro-oesophageal junction caused by vomiting, one of which was a Mallory-Weiss tear.

### Cohort 2: Balloon Placement Without Screening Endoscopy

#### Findings on Screening History

In cohort 2, the same detailed history as described previously was taken for each patient. Special attention was given to the details on history that came forward from cohort 1 (i.e. nausea, vomiting, belching, heartburn, history of hiatal hernia). No patient reported any gastrointestinal symptoms. The Orbera balloon was then placed without endoscopy, under fluoroscopic guidance.

#### Findings on Balloon Removal

All but three patients (94%) had an unremarkable balloon therapy period. Full endoscopic reports were available in 43 patients. Upon removal of the Orbera balloon, there were minimal findings on endoscopy (Table 5). Thirty-nine (90.7%) patients had no findings on endoscopy. Three patients did not tolerate the balloon. Of these, two patients had no abnormalities upon endoscopic examination during balloon removal. One was found to have a 4-cm hiatal hernia with grade B oesophagitis. Three balloon-tolerant patients had oesophagitis (two grade B; one grade C) without complaints, and two of these had a small hiatal hernia.

Upon removal, one major complication occurred. The grasper caught the oesophageal wall and upon removal of the balloon, an oesophageal tear occurred, subsequently causing an oesophageal perforation which had to be closed surgically.

### Complications of Balloon Treatment

Twenty-five patients in cohorts 1 and 2 had findings upon balloon removal or were intolerant of the balloon. We attempted to correlate intolerance and findings upon balloon removal to history, screening endoscopy, *Helicobacter pylori* status and findings on biopsies, oesophageal manometry, 24-h pH monitoring and gastric emptying time (data not shown). None of the findings was associated with the findings observed upon removal of the balloon.

## Discussion

A search of the literature shows that the majority of studies on intragastric balloon treatment report results of weight loss and changes in co-morbidities, but data on complaints, related endoscopic findings and their utility in screening out patients with contraindications are lacking [7]. The goal of this study was to evaluate the safety of placing balloons under fluoroscopic guidance without a previous endoscopy and to assess the utility of screening endoscopy to assess the eligibility of patients and its role in predicting adverse outcomes of balloon treatment.

In this study, both patients in cohort 1 who had contraindications had severe symptoms on history that correlated with findings on endoscopy of a hiatal hernia with grade D oesophagitis and a peptic ulcer, respectively. Indeed, in the 253 patients assessed in cohort 1, a history alone would have been adequate as a screening method, questioning the use of routine endoscopy for screening balloon patients. Moreover, in 57 patients in our study assessed with both screening endoscopy and X-ray with contrast, X-ray was actually more effective in identifying small anatomical abnormalities such as hiatal hernia. This result further questions whether endoscopy should be the first test performed if further work-up is required after taking a careful history.

As data on screening endoscopy before endoscopic bariatric therapy were lacking, we searched the literature on the use of endoscopy as a screening procedure in the field of bariatric surgery. In 2013, the American Society for Metabolic and Bariatric Surgery (ASMBS), The Obesity Society and the American Association of Clinical Endocrinologists published guidelines for the preoperative evaluation of a patient undergoing bariatric surgery that indicate a screening endoscopy is not part of a standard work-up, and gallbladder ultrasound and *H*. *pylori* screening is optional (recommendation grade D) [17]. In 2015, the American Society for Gastrointestinal Endoscopy in conjunction with the Society of Gastrointestinal and Endoscopic Surgeons and the ASMBS suggested that the decision to perform preoperative endoscopy should be individualised (low-quality evidence) [18]. Patients with symptoms of GORD or who use chronically H_2_ blockers or PPIs should have an upper GI endoscopic evaluation. Both guidelines preceded two recent meta-analyses which attempted to put the (routine) use of preoperative endoscopy into perspective [19, 20]. Both concluded that a routine endoscopy prior to bariatric surgery is not warranted as the incidence of significant findings, changing the type or timing of surgery, is low and a selective approach may be considered based on patients symptoms, risk factors and type of surgery planned. The results presented here would strongly support a similar recommendation for intragastric balloon placement.

Given these findings, we investigated whether balloons could be placed without endoscopy, using only a careful history as a screening tool, with emphasis on symptoms of severe nausea and vomiting, belching, heartburn and a history of hiatal hernia that were brought to the foreground by retrospective findings from cohort 1. We placed balloons in 50 patients under fluoroscopic guidance without a screening endoscopy. In 47 (94%) patients, there were no adverse events during balloon therapy. Three patients did not tolerate the balloon and in one, a large hiatal hernia and grade B oesophagitis was noted upon removal.

Could findings on a screening endoscopy predict balloon intolerance and complications? In our 151 patients who received a balloon after a screening endoscopy or under direct endoscopic visualisation and who had a follow-up endoscopy for balloon removal, this was not the case. None of the findings on endoscopy correlated to complications during balloon therapy. Even more sophisticated examinations such as manometry, 24-h pH measurements and gastric emptying studies were not helpful in predicting the outcome. Given our experience and the available literature, other than a careful history, the only intervention that appears to protect patients prior to balloon therapy is to begin acid suppression therapy. Indeed, Rossi et al. have shown that intragastric balloon therapy increases the risk of erosive oesophagitis and indicates that acid suppression therapy should be instituted prior to and during the therapy period [21]. While the mechanism for this increase is unclear, the increased rate of reflux and oesophagitis has previously been ascribed to increased transient LOS relaxations with a potential involvement of cholecystokinin-A receptors [14, 15].

Not only is the yield of endoscopy as a screening procedure for balloon therapy very low, it is expensive and not without risk. Obesity has previously been linked to an increased frequency of sedation-related adverse events, including cardiac and respiratory events, in patients undergoing advanced endoscopic procedures with propofol [22, 23]. Moreover, most practitioners prefer to remove balloons under general anaesthesia with tracheal intubation which is well known to be more risky in obese patients due to anatomical effects of fat deposition in the pharyngeal wall, altered pulmonary mechanics and reduced functional residual capacity [24]. As such, when possible, endoscopy should be avoided in obese patients, especially those undergoing an elective procedure like intragastric balloon therapy. Finally, serious adverse events can occur during the endoscopic removal of intragastric balloons, as seen in our experience. Specifically, oesophageal perforation, bleeding and aspiration pneumonitis have been described previously as known complications of endoscopic intragastric balloon removal [25].

The limitations of our study should be acknowledged. Although it was useful for standardisation across cohorts, these data come from one institution experienced in balloon therapy and from patients involved in previous clinical studies. The conclusions made in this study are limited by the small size of cohort 2, particularly in the setting of a rather low frequency of adverse events, as demonstrated in our large study on 815 patients treated in a private practice setting [26]. Also, our study was done in an area with a low incidence of *H*. *pylori* (19% in the present series) and a low incidence of upper gastrointestinal cancer.

## Conclusion

Our experience demonstrates that intragastric balloons may safely be placed without endoscopy using fluoroscopic guidance and a careful screening history prior to placement. Moreover, endoscopy prior to balloon placement does not predict complications during balloon therapy, and endoscopy is not without risk, particularly during intragastric balloon removal.
